# Optimizing stereotactic ablative body radiotherapy for ultra-central lung lesions: a comparative dosimetric analysis

**DOI:** 10.1186/s13014-025-02675-y

**Published:** 2025-06-18

**Authors:** Dan Tao, Lisi Sun, Lulu Wang, Lina Yang, Wei Zhou, Xiumei Tian, Xianfeng Liu

**Affiliations:** 1 Department of Radiation Oncology, Chongqing University Cancer Hospital, 181 Hanyu Road, Shapingba District, Chongqing, 400030 China; 2 Chongqing Key Laboratory of Translational Research for Cancer Metastasis and Individualized Treatment, Chongqing University Cancer Hospital, Chongqing, China

**Keywords:** Stereotactic ablative body radiotherapy, Lung lesions, Ultra-central, Helical tomotherapy, Volumetric modulated arc therapy

## Abstract

**Background:**

Stereotactic ablative body radiotherapy (SABR) for ultra-central (UC) lung tumors is still challenging due to potentially severe complications caused by overdoses to critical mediastinal structures. This study aimed to compare the dosimetric advantages between volumetric modulated arc therapy (VMAT)-SABR and Helical Tomotherapy (HT)-SABR in UC lung lesions.

**Methods:**

From August 14, 2019, to June 4, 2023, patients with ultra-central (UC) lung tumors who received SABR for lung tumors were enrolled in this study. Two different radiotherapy plans were created based on the CT datasets of each case on the Eclipse and Tomotherapy treatment planning systems respectively. All patients received 60 Gy in 8 fractions. The parameters for dose evaluation of the target volumes and organs at risk (OARs) were compared based on the data extracted from dose-volume histograms. All of the statistical analyses were performed with SPSS 17.0 software.

**Results:**

Compared with HT-SABR, VMAT-SABR significantly increased the D_mean_ of the iGTV (*p* = 0.002) and the D_mean_, D_10%_, and D_5%_ of the PTV (*p* = 0.000, 0.000, and 0.002, respectively). The values of D_2cm_, 95% isodose volume, and GI of VMAT-SABR significantly decreased by 15.11%, 7.78%, and 20.65% (*p* = 0.000; 0.000; 0.000) respectively. The values of D_max_ of the spinal cord, spinal cord + 3 mm, esophagus, heart, trachea, proximal bronchial tree (Pbtree), and great vessels, and D_mean_ of the Non-GTV Lung and ipsilateral Non-GTV Lung with VMAT-SABR were significantly lower than those of HT-SABR.

**Conclusion:**

Our study revealed that VMAT-SABR showed a steeper dose falloff and lower dose of the OARs. VMAT-SABR is a potentially optimal modality to improve the therapeutic ratio compared to HT-SABR in patients with UC lung lesions.

## Introduction

Lung cancer is the leading cause of global cancer incidence and mortality [1], and stereotactic ablative body radiotherapy (SABR) has become the primary treatment strategy for inoperable early-stage lung cancer [2]. For peripherally located, medically inoperable early-stage non-small cell lung cancer (NSCLC), SABR has been accepted as standard-of-care [3]. However, centrally located lung tumors were historically considered a “no-fly zone” of SABR due to the potential risk of severe radiotherapy-induced toxicity, particularly massive hemoptysis [4]. The prospective multi-institutional clinical trial RTOG 0813 revealed acceptable toxicity with 5-fraction SABR [5]. Several other SABR regimens such as 50 Gy in four fractions, and 60 Gy in eight fractions, also have been shown to be safe and well tolerated in patients with centrally located lung tumors [6, 7].

Ultra-central (UC) lung tumors are a special subtype of central lung tumors, which are defined as tumors that are in extreme proximity to the central airway, esophagus, heart, and great vessels [8]. However, up to date, UC lung lesions remain a “black box” for radiotherapists and are still largely unexplored territory, despite that RTOG 0813 has established the feasibility of SABR in central lung tumors. Over the past decade, several retrospective studies have shown promising results regarding both the efficacy and safety of SABR in UC lung lesions and several SABR schemes have shown satisfied local control and overall survival [8, 9]. However, the reported incidence of grade 5 hemoptysis was found to be as high as 15%, which presents a significant challenge for the application of SABR in UC lung lesions [10, 11]. Although varieties of modalities are available for SABR, there is currently no consensus on the optimal technique due to its requirement for precise conformity and rapid dose gradient. The most commonly employed SABR modalities include fixed-field intensity modulated radiotherapy (IMRT) and volumetric modulated arc therapy (VMAT), while Cyberknife Robotic Radiosurgery and Helical Tomotherapy (HT) are also frequently implemented in certain centers. However, the optimal modalities for SABR in the treatment of patients with UC lung lesions are still unclear.

Hence, the objective of this study was to compare the dosimetric advantages between VMAT-SABR and HT-SABR in UC lung lesions.

## Materials and methods

### Patients selection

**Fig. 1:**
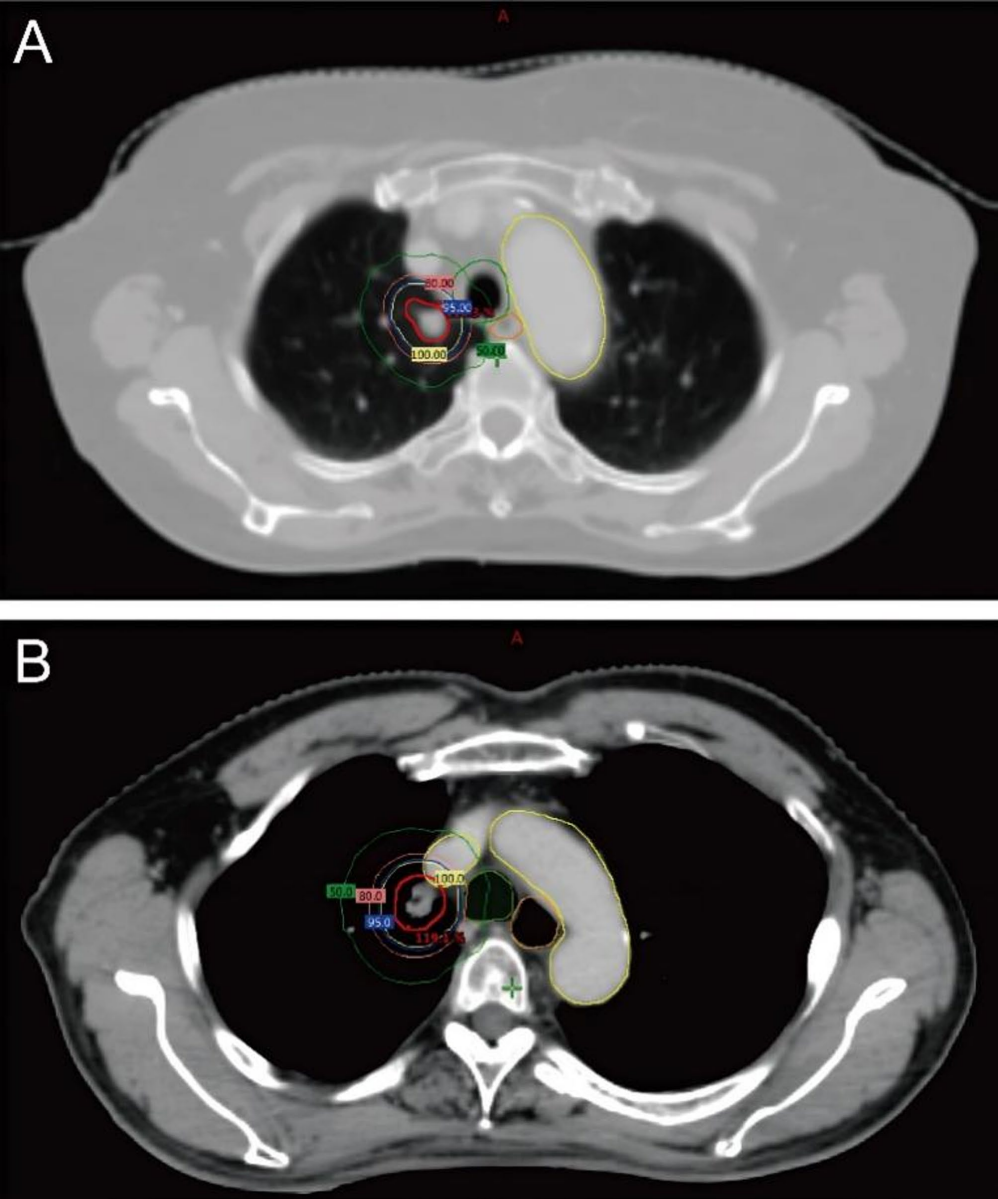
Representative cases where (**A**) GTV is closely contacting trachea (**B**) GTV is closely contacting superior vena cava. Abbreviations: GTV, gross tumor volume

From August 14, 2019, to June 4, 2023, patients who received SABR for lung tumors were screened. The inclusion criteria include: (1) lung tumor located ultra-centrally; (2) patients suitable for SABR (tumor size ≤ 5 cm). Exclusive criteria include: (1) lung tumor located peripherally or centrally; (2) patients were not suitable for SABR due to size of tumors (> 5 cm). The definition of ultra-centrally located lung tumors was tumors with planning target volume (PTV) directly abutting or overlapping with the proximal bronchial tree (Pbtree), trachea, esophagus, heart, or great vessels. Representative cases where the PTV is directly abutting the trachea and great vessels are shown in Fig. 1. This study was approved by the Ethics Committee of the Chongqing University Cancer Hospital. The Institutional Ethics Committee waived the need for informed consent due to the retrospective nature of this study.

### Immobilization and simulation

All patients were positioned in a supine position and immobilized using a polyurethane styrofoam and thermoplastic mask, with their arms placed above the head. The 4D-CT scan was acquired for each patient using a Philips Big Bore CT scanner (Philips, Amsterdam, Netherlands) with a slice thickness of 3 mm and contrast enhancement. The scan range extended from the mandible to the thorax, encompassing all organs at risk (OARs) including the esophagus, heart, trachea, proximal bronchus, lungs, and great vessels. The images were transferred to the Eclipse treatment planning system (TPS, Version 15.6, Varian Medical Systems, Inc.) for contouring and planning purposes.

### Definition of target volumes and oars

The target volumes and adjacent normal tissues were contoured on the Eclipse TPS. The internal gross tumor volume (iGTV) was delineated on the maximum intensity projection (MIP) datasets and rigidly projected onto the average intensity projection (AIP) image of 4D-CT. No clinical target volume (CTV) expansion was utilized (i.e. CTV = GTV). An isotropic 5 mm expansion was applied to the iGTV to generate the PTV. The adjacent OARs, including the ipsilateral brachial plexus, trachea, Pbtree, esophagus, great vessels, non-GTV lung (a virtual structure defined as the remaining lung tissue excluding the GTV), ipsilateral non-GTV lung, spinal cord, and a virtual organ (spinal cord + 3 mm: obtained by uniformly extending the spinal cord in all directions for a distance of 3 mm), were meticulously contoured layer by layer on the AIP datasets in the Eclipse TPS. Subsequently, the AIP datasets, along with the corresponding contoured organs, were transferred to the Tomotherapy TPS (Version 2.1.9, Accuray, Inc.).

### Radiotherapy plans

Two distinct radiotherapy plans were created based on the CT datasets of each of the twenty-five cases, utilizing the Eclipse and Tomotherapy TPS, respectively. The plans employed 6 MV photon beams generated by the Edge device (Varian, USA) and the latest upgraded version of Tomotherapy treatment units (Accuray, USA) correspondingly.

#### VMAT-SABR plans

The VMAT-SABR plans were designed using the Eclipse TPS. Each plan consisted of three 180° arcs on the affected side (181°-0° or 0°-179° round-trip rotation). The collimator underwent a rotation of 30° or 330°, while the treatment couch underwent rotations of 0°, 345°, and 15°, respectively. The isocenter of the three arcs coincided with the geometric center of the target volume. The SRS-ARC technology and energy 6X-FFF were adopted for optimization, while the acuros external beam algorithm was utilized for final dose calculations in all plans.

#### HT-SABR plans

The HT-SABR plans were designed utilizing the Tomotherapy TPS, with optimization performed with a modulation factor of 3, a pitch value of 0.156, and a jaw width of 2.5 cm. Simultaneously, all plans employed fine grids and dynamic jaw mode for optimization.

The prescribed dose was set as 60 Gy in 8 fractions. For all plans, the prescription dose should encompass a minimum of 95% of the PTV, and at least 90% of the prescription dose should be delivered to 99% of the PTV. Additional dose constraints for target volumes were based on previous reports [11]. All plans were normalized so that the prescription dose covers 95% of the PTV. The dose constraints for OARs were tabulated in Table 1. D_max_ represented the maximum dose received by the structure, while D_mean_ indicated the mean dose of the organ. Additionally, D_5cc_ and D_10cc_ denoted the doses received by a volume of 5cm^3^ and 10cm^3^ within the corresponding organ respectively, whereas V_20Gy_ signified the volume of that particular organ receiving ≥ 20 Gy.

**Table 1:** Dose constrains of OARs

Structures	Parameters	Constrains
Spinal Cord	D_max_	28 Gy
Spinal Cord + 3 mm	D_max_	32 Gy
Esophagus	D_max_	45 Gy
	D_5cc_	40 Gy
Ipsilateral Brachial Plexus	D_max_	30 Gy
Heart	D_max_	64 Gy
	D_10cc_	60 Gy
Trachea	D_max_	64 Gy
	D_10cc_	60 Gy
Pbtree	D_max_	64 Gy
	D_10cc_	60 Gy
Non-GTV Lung	V_20Gy_	10–15%
	D_mean_	< 12 Gy
Great Vessels	D_max_	64 Gy
	D_10cc_	60 Gy
Ipsilateral Non-GTV Lung	V_20Gy_	
	D_mean_	

Abbreviations: OARs, organs at risk; D_max_, the maximum dose of the structure; D_xxcc_, dose covering xx cubic centimeter of the structure; Pbtree, proximal bronchial tree; Non-GTV Lung, a virtual structure created by subtracting the gross tumor volume from the lung volume; V_20Gy_, the percentage volume of the structure receiving ≥ 20 Gy; D_mean_, the mean dose of the structure

### Dose evaluation for the target volumes and normal tissues

**Fig. 2:**
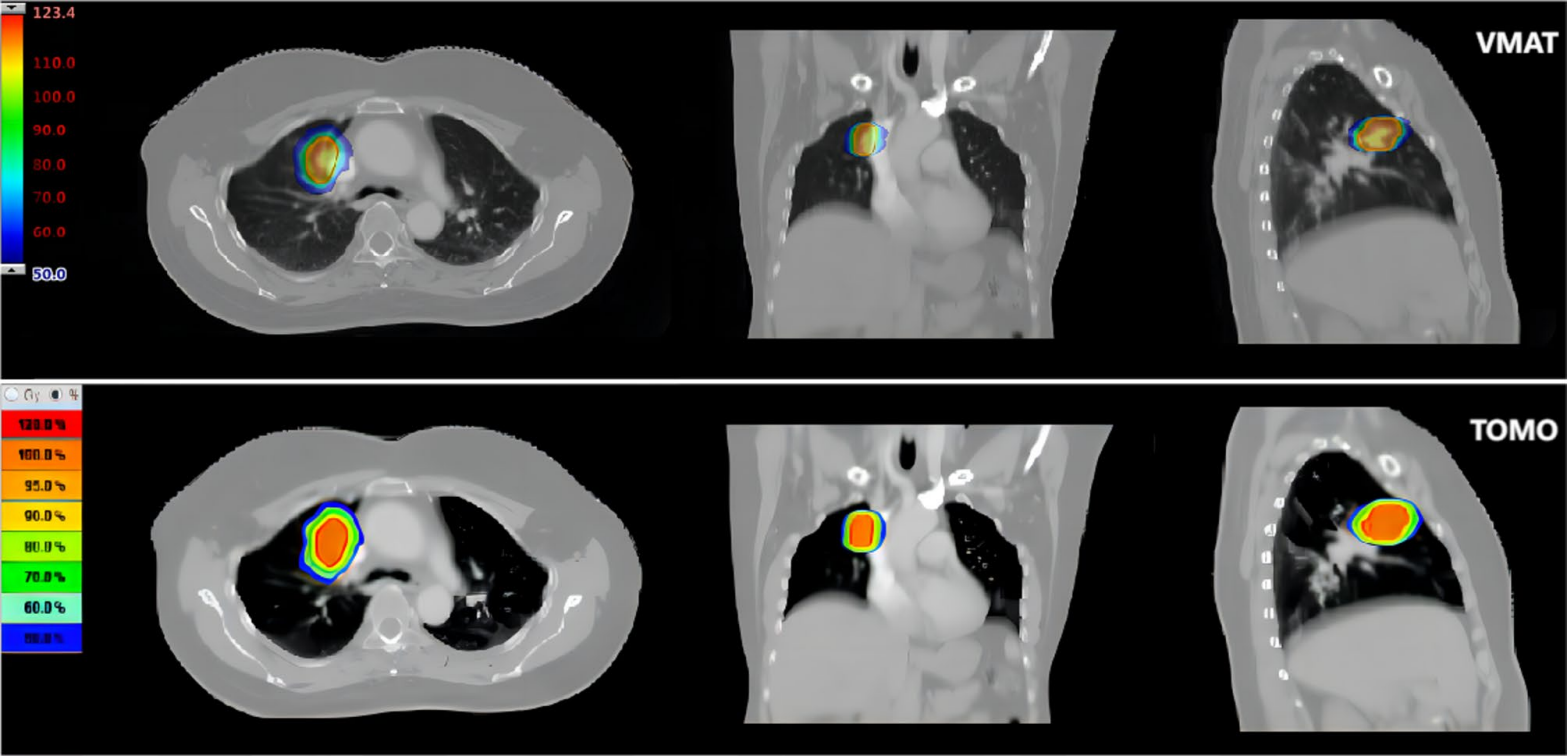
Illustration of the dose distribution utilizing VMAT and TOMO techniques for a single patient. Abbreviations: VMAT, volumetric modulated arc therapy; TOMO, Helical Tomotherapy

**Fig. 3:**
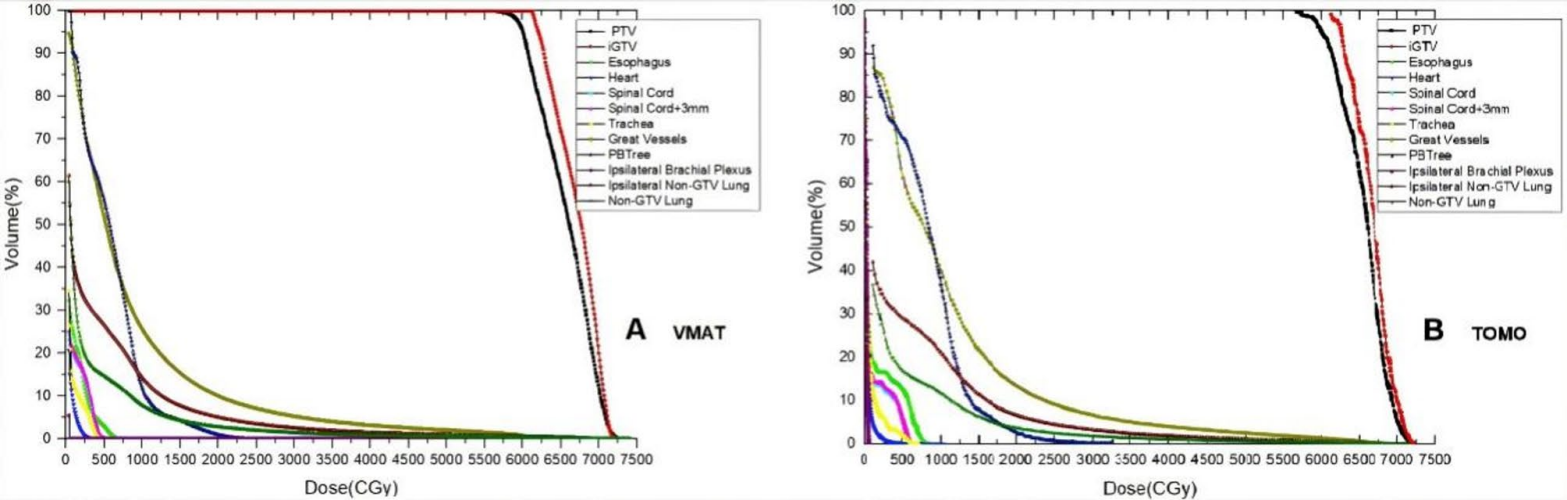
DVHs for (**A**) VMAT and (**B**) TOMO plans for a single patient. Abbreviations: DVHs, Dose-Volume Histograms; VMAT, volumetric modulated arc therapy; TOMO, Helical Tomotherapy

The data derived from dose-volume histograms (DVHs) of the fifty plans were analyzed in this study. Figures 2 and 3 showed representative dose distribution and DVHs for the two types of modalities, respectively. The dose evaluation parameters for target volumes primarily included the subsequent metrics.

iGTV Coverage: D_mean_, D_max_, and D_min_ (representing the minimum dose).

PTV Coverage: V_95%_ (the volume of PTV covered by ≥ 95% of the prescribed dose), D_mean_, and the following four parameters of D_99%_, D_10%_, D_5%_, and D_1%_ (dose covering 99%, 10%, 5% and 1% of the PTV, respectively).

D_2cm_(%): the D_max_ at any direction 2 cm away from the PTV, expressed as a percentage of the prescribed dose.

95% Isodose Volume: the coverage volume of 95% isodose line.

The Paddick conformity number (CN): the Paddick CN was calculated as follows:

1 $$\:\text{P}\text{a}\text{d}\text{d}\text{i}\text{c}\text{k}\:\text{C}\text{N}=\frac{{\left({\text{T}\text{V}}_{\text{P}\text{I}\text{V}}\right)}^{2}}{\left(\text{T}\text{V}\text{*}\text{P}\text{I}\text{V}\right)}$$

where TV_PIV_ represented the target volume covered by the 100% isodose line, while TV was the target volume, and PIV denoted the volume covered by the 100% isodose line.

The uniformity index (UI): the UI was calculated by:

2 $$\:\text{U}\text{I}=\frac{{\text{D}}_{5\text{\%}}}{{\text{D}}_{95\text{\%}}}$$

where D_5%_, and D_95%_ were the dose received by 5% and 95% of the PTV volume, respectively.

The heterogeneity index (HI): the HI was determined by:

3 $$\:\text{H}\text{I}=\frac{{\text{D}}_{10\text{\%}}}{{\text{D}}_{95\text{\%}}}$$

where D_10%_, and D_95%_ were the dose to the hottest 10% of the PTV and the dose to the 95% of the PTV coverage respectively.

The gradient index (GI): the GI was given by:

4 $$\:\text{G}\text{I}=\frac{{\text{V}}_{50\text{\%}}}{{\text{V}}_{100\text{\%}}}$$

where V_50%_, and V_100%_ were the absolute volumes covered by 50% and 100% isodose lines, respectively.

The D_max_ of the spinal cord, spinal cord + 3 mm, esophagus, ipsilateral brachial plexus, heart, trachea, Pbtree, and great vessels were evaluated. Additionally, the D_5cc_ of the esophagus and the D_10cc_ of the four organs heart, trachea, Pbtree and great vessels were assessed. Furthermore, the D_mean_ and V_20Gy_ of Non-GTV Lung and ipsilateral Non-GTV Lung mainly were also analyzed.

### Statistical analysis

All statistical analyses were carried out using SPSS 17.0 software (SPSS, Chicago, IL, USA). To determine the statistical significance of our results, a paired Student t-test was used to compare the dosimetric differences of target volumes and normal tissues in patients treated with the 2 strategies. The value of *P* less than 0.05 indicated that the difference was statistically significant.

## Results

### Characteristics of the enrolled patients

Twenty-five patients meeting the criteria for UC lung tumors were enrolled in this study, including both primary and metastatic lesions. The cohort comprised 14 male and 11 female patients, aged 33–83 years at the time of radiotherapy. Tumor characteristics included 22 primary lesions, 2 metastatic lesions, and 1 recurrent lesion. The presence of metastatic lesions in our cohort reflects the clinical reality that UC lung tumors may arise through either primary development or secondary spread to this critical anatomical region. Detailed clinicopathological characteristics are presented in Table 2.

**Table 2:** Clinicopathological characteristics of patients with ultra-central lung lesions

Case	Gender	Age(year)	ECOG PS	Tumor type	Tumor histology	PTV abutting/overlap with OAR
Case 1	Female	47	0	Primary tumor	Lung adenosquamous carcinoma	Proximal bronchial tree
Case 2	Female	54	0	Primary tumor	Small cell lung cancer	Proximal bronchial tree
Case 3	Female	81	0	Primary tumor	Lung adenocarcinoma	Trachea
Case 4	Male	51	0	Primary tumor	Lung adenocarcinoma	Great vessel
Case 5	Male	33	0	Metastatic tumor	Prostate carcinoma	Heart
Case 6	Male	65	0	Primary tumor	Lung adenocarcinoma	Trachea
Case 7	Female	60	0	Primary tumor	Lung adenocarcinoma	Great vessel
Case 8	Male	57	0	Primary tumor	Lung squamous cell carcinoma	Proximal bronchial tree
Case 9	Female	48	0	Primary tumor	Lung adenocarcinoma	Great vessel
Case 10	Male	66	0	Recurrent tumor	Lung adenocarcinoma	Proximal bronchial tree
Case 11	Female	54	2	Primary tumor	Lung adenocarcinoma	Proximal bronchial tree
Case 12	Female	56	1	Primary tumor	Lung adenocarcinoma	Great vessel
Case 13	Male	72	1	Primary tumor	Lung adenocarcinoma	Great vessel
Case 14	Male	76	0	Primary tumor	Lung squamous cell carcinoma	Trachea
Case 15	Male	79	0	Primary tumor	Lung adenocarcinoma	Heart
Case 16	Female	83	1	Primary tumor	Lung adenocarcinoma	Heart
Case 17	Female	58	2	Primary tumor	Lung adenocarcinoma	Great vessel
Case 18	Male	50	1	Primary tumor	Lung adenocarcinoma	Great vessel
Case 19	Male	78	1	Primary tumor	Lung adenocarcinoma	Proximal bronchial tree
Case 20	Male	78	0	Primary tumor	Lung adenocarcinoma	Great vessel
Case 21	Male	58	0	Primary tumor	Lung adenocarcinoma	Great vessel
Case 22	Male	80	0	Primary tumor	Lung adenocarcinoma	Proximal bronchial tree
Case 23	Male	83	1	Primary tumor	Lung adenocarcinoma	Heart
Case 24	Female	57	0	Metastatic tumor	Renal cell carcinoma	Proximal bronchial tree
Case 25	Female	42	2	Primary tumor	Small cell lung cancer	Proximal bronchial tree

Abbreviations: PS, performance score; PTV, planning target volume; OAR, organ at risk

### Dose evaluation for the target volumes

The dosimetric results for the target volumes are presented in Table 3. Compared to HT-SABR, VMAT-SABR significantly increased the D_mean_ of the iGTV (*p* = 0.002) and the PTV (*p* < 0.001), as evidenced by increases in D_10%_(*p* < 0.001) and D_5%_ (*p* = 0.002). Additionally, VMAT-SABR resulted in significant reductions in D_2cm_, 95% isodose volume, and GI by 15.11%, 7.78%, and 20.65% (all *p* < 0.001) compared to HT-SABR. Conversely, the Paddick CN, UI, and HI of VMAT-SABR exhibited slight increases of 3.30%, 1.74%, and 1.77%, respectively.

**Table 3:** Dosimetric comparison results of target volume

Structures	Parameters	VMAT-SABR	HT-SABR	*p*-Value
iGTV	D_mean_(%)	111.40 ± 2.67	109.19 ± 3.04	0.002
	D_max_(%)	120.73 ± 2.42	120.52 ± 2.21	0.668
	D_min_(%)	100.6 ± 3.92	101.48 ± 2.90	0.135
PTV	V_95%_(cc)	51.75 ± 45.62	51.76 ± 45.51	0.774
	D_mean_(%)	109.06 ± 1.21	107.38 ± 1.45	0.000
	D_99%_ (%)	96.87 ± 0.39	97.05 ± 0.72	0.238
	D_10%_(Gy)	69.21 ± 1.03	67.99 ± 1.33	0.000
	D_5%_(Gy)	69.92 ± 0.96	68.99 ± 1.35	0.002
	D_1%_(%)	118.26 ± 1.56	117.72 ± 2.14	0.222
	D_2cm_(%)	45.24 ± 7.77	53.29 ± 9.73	0.000
	95% Isodose Volume	55.86 ± 48.54	60.57 ± 50.48	0.000
	Paddick CN	0.94 ± 0.01	0.91 ± 0.02	0.000
	UI	1.17 ± 0.02	1.15 ± 0.02	0.002
	HI	1.15 ± 0.02	1.13 ± 0.02	0.000
	GI	3.42 ± 0.32	4.31 ± 0.65	0.000

Abbreviations: iGTV, internal gross tumor volume; D_mean_, the mean dose of the target volume; D_max_, the maximum dose of the target volume; D_min_, the minimum dose of the target volume; PTV, planning target volume; V_95%_, the volume of target volume covered by ≥ 95% of the prescribed dose; D_xx%_, dose covering xx% the target volume; D_2cm_, the maximum dose at any direction 2 cm away from the target volume; CN, conformation number; UI, uniformity index; HI, heterogeneity index; GI, gradient index

### Dose evaluation for the oars

The dosimetric parameters for the OARs are summarized in Table 4. Compared to HT-SABR, VMAT-SABR resulted in a significant reduction in D_max_ of spinal cord, spinal cord + 3 mm, esophagus, heart, trachea, Pbtree, and great vessels by 28.86%, 27.91%, 25.06%, 21.30%, 11.43%, 24.79%, and 11.61% (*p* = 0.000; 0.000; 0.000; 0.001; 0.004; 0.000; 0.000), respectively. Additionally, the D_5cc_ of the esophagus and the D_10cc_ of the heart, trachea, Pbtree, and great vessels with VMAT-SABR were significantly reduced by 18.68%, 23.40%, 20.94%, 28.41%, and 18.71% (*p* = 0.002; 0.001; 0.006; 0.006; 0.000), respectively. Furthermore, the D_mean_ of the Non-GTV Lung and ipsilateral Non-GTV Lung with VMAT-SABR were significantly decreased by 9.01% and 7.17% (*p* < 0.001), respectively.

**Table 4:** Dosimetric comparison results of OARs

Structures	Parameters	VMAT-SABR	HT-SABR	*p*-Value
Spinal Cord	D_max_(Gy)	9.07 ± 4.73	12.75 ± 5.20	0.000
Spinal Cord + 3 mm	D_max_(Gy)	10.95 ± 6.22	15.19 ± 7.28	0.000
Esophagus	D_max_(Gy)	17.40 ± 6.69	23.22 ± 11.04	0.000
	D_5cc_(Gy)	8.40 ± 5.91	10.33 ± 7.06	0.002
Ipsilateral Brachial Plexus	D_max_(Gy)	7.05 ± 12.9	7.45 ± 16.16	0.763
Heart	D_max_(Gy)	20.69 ± 22.81	26.29 ± 25.97	0.001
	D_10cc_(Gy)	12.11 ± 13.63	15.81 ± 17.5	0.001
Trachea	D_max_(Gy)	14.65 ± 16.8	16.54 ± 18.68	0.004
	D_10cc_(Gy)	4.87 ± 7.07	6.16 ± 8.57	0.006
Pbtree	D_max_(Gy)	23.03 ± 22.87	30.62 ± 23.65	0.000
	D_10cc_(Gy)	3.78 ± 4.18	5.28 ± 6.06	0.006
Great Vessels	D_max_(Gy)	36.07 ± 21.89	40.81 ± 22.31	0.000
	D_10cc_(Gy)	15.25 ± 10.54	18.76 ± 12.92	0.000
Ipsilateral Non-GTV Lung	V_20Gy_(%)	10.88 ± 7.12	11.67 ± 6.42	0.261
	D_mean_(Gy)	7.12 ± 3.80	7.67 ± 3.81	0.000
Non-GTV Lung	V_20Gy_(%)	5.27 ± 3.36	6.58 ± 5.11	0.051
	D_mean_(Gy)	4.34 ± 2.24	4.77 ± 2.23	0.000

Abbreviations: OARs, organs at risk; D_max_, the maximum dose of the structure; D_xxcc_, dose covering xx cubic centimeters of the structure; Pbtree, proximal bronchial tree; Non-GTV Lung, a virtual structure created by subtracting the gross tumor volume from the lung volume; V_20Gy_, the volume of the structure receiving ≥ 20 Gy; D_mean_, the mean dose of the structure

## Discussion

SABR delivers a large dose of radiation in fewer fractions to tumor targets with superior conformality, precision, dose gradient, and efficiency compared with conventionally fractionated radiotherapy. Due to the potential dosimetric advantages of SABR, it has emerged as a prominent and extensively discussed treatment modality for central or UC lung lesions in recent years. Currently, the optimal modality for achieving both efficacy and safety in SABR for treating UC lung lesions remains unresolved, despite the availability of various modalities such as fixed-field IMRT, VMAT, HT, and Cyberknife [12]. Therefore, it is imperative to investigate the optimal radiotherapy modality for SABR in UC lung lesions treatment. This study unequivocally demonstrates that VMAT-SABR surpasses HT-SABR in terms of various plan evaluation metrics, including conformality index, dose gradient, and OARs sparing. To the best of our knowledge, this is the first study to explore optimal modalities of SABR for UC lung lesions.

VMAT has emerged as a widely adopted modality for delivering SABR due to its superior dose distribution advantages and treatment efficiency compared to fixed-field IMRT [13]. However, effectively sparing critical OARs, such as the trachea, bronchus, esophagus, heart, and great vessels, remains a significant challenge in VMAT for the treatment of central or UC lung lesions [14–17]. Loi et al. [18] previously reported 5 cases (5%) of severe toxicity (grade ≥ 3) including radiation pneumonitis (RP), hemoptysis, bronchial stricture and radiation esophagitis among 109 patients with UC lung oligometastases treated with VMAT-SABR (median BED10 = 105.0 Gy10, range 75.0–132.0 Gy10, 6–10 Gy/fraction). Therefore, it is urgent to further optimize the VMAT-SABR technique to minimize radiation-induced toxicity in the treatment of UC or to explore alternative treatment modalities.

HT involves the continuous rotation of the gantry by 360° around the patient, while simultaneously translating the couch through the gantry bore. The beam intensity is dynamically modulated throughout this process to precisely conform to the target volume and a built-in megavoltage CT (MVCT) image will be acquired before each treatment fraction. Numerous dosimetric studies have consistently demonstrated that HT exhibits superior conformality index, target volume coverage, and normal tissue sparing compared to both step-and-shoot IMRT and VMAT, particularly in complex cases such as advanced T-stage head and neck cancers where lesions are adjacent to multiple OARs [19]. Moreover, dose distribution achieved by HT tends to be more homogeneous [20–22]. Hence, HT should be qualified to carry out SABR [23]. In 2006, Hodge et al. demonstrated the technical feasibility of MVCT-guided SABR with HT in medically inoperable early-stage lung cancer [24]. Subsequent studies have also reported encouraging results applying HT-SABR (48–70 Gy/3–10 F) to early NSCLC or lung metastasis with a favorable efficacy and moderate toxicity [25–32]. In 2011, Chi, A. et al. published the first dosimetric study validating the feasibility of HT-SABR (70 Gy/10F) in 10 central lung tumors [26]. The local control, overall survival, and toxicity seemed to be favorable with HT-SABR treating central lung tumors [22, 31, 33].

The VMAT and HT techniques are renowned for their exceptional ability to deliver high-quality dose coverage of the target volume. Nevertheless, considering the complex spatial relationship between the target volume and adjacent OARs, it is still worth studying whether these two techniques can maintain their dosimetric advantages in treating central or UC lung lesions. This study found similar PTV coverage with HT-SABR and VMAT-SABR, as shown by V_95%_ (51.75 ± 45.62 vs. 51.76 ± 45.51, *p* = 0.774). Chi, A et al., and Kannarunimit et al. [34] also reported similar results in their cohort of patients with central lung cancer. A previous study demonstrated better conformality with VMAT than HT when delivering conventionally fractionated radiotherapy to lung cancers [35]. The results of our study showed that VMAT achieved a better Paddick CN compared to HT, supporting a superior conformality in SABR for patients with UC lung tumors. Both the two studies mentioned above found equivalent conformality between the two modalities during SABR of central lung cancers [34, 36], even when more arcs were added during VMAT planning [36].

This study observed superior dose uniformity achieved with HT-SABR compared to VMAT-SABR (HI 1.15 ± 0.02 vs. 1.13 ± 0.02, *p* = 0.000; UI 1.13 ± 0.02 vs. 1.15 ± 0.02, *p* = 0.000). Increasing the number of arcs has been shown to improve dose homogeneity inside the target volume [36, 37]. However, in this study, routine use of a 3-arc VMAT is considered sufficient for most clinical scenarios as increasing the number of arcs would unnecessarily prolong treatment time per fraction. Besides, dose homogeneity is not the primary consideration for SABR planning. Otherwise, rapid dose drop-off is imperative to spare the normal organs surrounding central or UC lung lesions due to their close anatomical relationship. Chi et al. [26] demonstrated the efficacy of HT by a remarked dose reduction ranging from 29 to 82% between the PTV and adjacent central normal structures while maintaining adequate PTV dose coverage and adhering to OAR constraints. However, these findings indicated that VMAT achieved a faster dose drop-off than HT, as evidenced by the lower D_2cm_ and GI values.

Generally, the available techniques for performing SABR exhibit subtle differences with regard to dose coverage and conformality. The distinguishing factor lies in their capability to spare the OARs. Consistent with several previous studies [13, 34, 36], we have found the application of VMAT resulted in a significant reduction in D_mean_ of both the non-GTV Lung and ipsilateral non-GTV Lung, which are metrics associated with the risk of clinically significant radiation-induced lung injury [38]. While other studies claimed that HT-SABR effectively protects normal lung tissue [24, 31, 32]. Aibe et al. [30] previously documented a 6.7% incidence rate of grade 5 RP after lung HT-SABR (29 of the 31 lung lesions were peripherally located), although both G5 RP cases had severe comorbidities before SABR. Therefore, HT-SABR appears to have little advantage over VMAT-SABR in terms of lung sparing. This is inherently attributed to the dynamic spiral modulation mechanism of HT, which results in low-dose spillage and an increased mean dose to the normal lung [13].

In the present study, VMAT demonstrated superior performance over HT in sparing most mediastinal structures, including the spinal cord, esophagus, heart, trachea, and Pbtree. This finding is consistent with the previous study that compared these two techniques in conventionally fractionated radiotherapy in locally advanced NSCLC and SABR in early-stage NSCLC [13, 35]. Chi et al. [36] found that HT or VMAT with more arcs showed better OAR sparing than 2-arc VMAT primarily for central lesions with 2 or more adjacent normal structures.

The dosimetric advantages observed with VMAT-SABR, including improved target conformity and sharper dose gradients, may have important clinical implications. While our study did not collect CTCAE-graded toxicity data, the reduced radiation exposure to adjacent organs at risk observed in our dosimetric analysis suggests a potential for decreased treatment-related toxicities, which could theoretically improve patient tolerance. The enhanced dose conformity achieved with VMAT-SABR may also contribute to more precise tumor targeting, though the clinical impact on local control and survival outcomes would require prospective validation with clinical endpoints. These dosimetric characteristics position VMAT-SABR as a technically advanced approach for UC lung tumors, but we emphasize that the observed advantages in organ sparing should be interpreted cautiously until confirmed by clinical toxicity data in future studies.

There are several shortcomings in this study. Firstly, as a purely dosimetric study, our investigation is constrained by the absence of clinical data that could provide insights into treatment efficacy and toxicity. Secondly, subgroup analysis has not been conducted to account for the potential influence of distinct clinical scenarios on determining the optimal technique of SABR. Kannarunimit et al. [34] proposed that central lung tumors could be categorized by PTV size and degree of PTV overlap with central structures. For patients with large PTV-OAR overlap (> 10% of PTV volume), 2-full arc VMAT and HT produced greater homogeneity than Robotic Radiosurgery (RR), and VMAT seemed to yield the lowest RP risk for a large PTV. RR might result in the lowest lung dose and dose falloff for patients with small PTV-OAR overlap (< 10% of the PTV volume) and small PTV. Thus, further investigation is warranted to elucidate the criteria for selecting an appropriate SABR modality for central or UC lung lesions. Additionally, the lack of consensus on diverse definitions, various regimens (e.g. 60 Gy/8f, 60 Gy/5f, 56 Gy/7f), and inconsistent OAR constraints for UC lung lesions across different countries and medical centers [9, 39], have compromised the generalizability of our study. Notwithstanding these limitations, the findings of this study can serve as a valuable reference for clinical practice to overcome the challenge of SABR for UC lung tumors.

## Conclusions

With the advancement of radiotherapy, investigations into the applicability of SABR for UC lung lesion treatment are on the rise. Studies comparing the dosimetric advantages between HT- and VMAT-SABR for UC lung lesions are scarce. This dosimetric study conducted on 25 patients with UC NSCLC showed that VMAT-SABR showed a steeper dose falloff and lower dose to the OARs. VMAT-SABR was associated with improved dosimetric sparing of organs at risk compared with HT-SABR, suggesting a potential reduction in toxicity risk for patients with UC lung lesions. Further investigations incorporating long-term follow-up of both survival and prospectively collected toxicity data are needed to establish clinical correlations with the identified dosimetric advantages.

## Data Availability

No datasets were generated or analysed during the current study.
